# Ethnic Differences in the Risk Factors and Severity of Coronary Artery Disease: a Patient-Based Study in Iran

**DOI:** 10.1007/s40615-017-0408-3

**Published:** 2017-08-03

**Authors:** Seyed Hesameddin Abbasi, Örjan Sundin, Arash Jalali, Joaquim Soares, Gloria Macassa

**Affiliations:** 1 Department of Health Sciences, Section of Public Health Sciences, Mid Sweden University, Mittuniversitetet, Campus Sundsvall, Storgatan 73, 851 70 Sundsvall, Sweden; 2 Tehran Heart Center, Tehran University of Medical Sciences, North Kargar Street, Tehran, 1411713138 Iran; 3 Department of Psychology, Mid Sweden University, 83125 Östersund, Sweden; 4 Department of Occupational and Public Health Sciences, University of Gävle, Kungsbäcksvägen 47, Building 55 (TOR), 4th floor, Gävle, Sweden

**Keywords:** Health status disparities, Ethnicity, Coronary artery disease, Iran

## Abstract

**Background:**

Diverse ethnic groups may differ regarding the risk factors and severity of coronary artery disease (CAD). This study sought to assess the association between ethnicity and CAD risk and severity in six major Iranian ethnic groups.

**Methods:**

In this study, 20,165 documented coronary artery disease patients who underwent coronary angiography at a tertiary referral heart center were recruited. The demographic, laboratory, clinical, and risk factor data of all the patients were retrieved. The Gensini score (an indicator of CAD severity) was calculated for all, and the risk factors and severity of CAD were compared between the ethnical groups, using adjusted standardized residuals, Kruskal–Wallis test, and multivariable regression analysis.

**Results:**

The mean age of the participants (14,131 [70.1%] men and 6034 [29.9%] women) was 60.7 ± 10.8 years. The Fars (8.7%) and Gilak (8.6%) ethnic groups had the highest prevalence of ≥4 simultaneous risk factors. The mean Gensini score was the highest for the Gilaks (77.1 ± 55.9) and the lowest among the Lors (67.5 ± 52.8). The multivariable regression analysis showed that the Gilaks had the worst severity (*β* 0.056, 95% CI 0.009 to 0.102; *P* = 0.018), followed by the Torks (*β* 0.032, 95% CI 0.005 to 0.059; *P* = 0.020). Meanwhile, the Lors showed the lowest severity (*β* −0.087, 95% CI −0.146 to −0.027; *P* = 0.004).

**Conclusions:**

This study found that there was heterogeneity in CAD severity and a diverse distribution in its well-known traditional risk factors among major Iranian ethnic groups.

## Introduction

Coronary artery disease (CAD) is considered the leading cause of death in most parts of the world, including Iran [1]. The bulk of CAD burden is allied to modifiable risk factors. Meanwhile, there are substantial ethnical/racial disparities both in the frequency of these CAD risk factors and in the severity of the disease [2–4]. Different ethnic groups are predisposed to develop CAD at different rates, giving rise to a higher prevalence or severity of the disease in certain populations. This disparity has been shown in both developed [2–5] and developing [6–8] countries.

Iran, a middle-income country located in the Middle East, is considered a multi-ethnic country. Fars, Torks, Kords, Gilaks, Mazanis, and Lors constitute the major ethnic groups of Iran. There are also some other minor ethnic groups such as Turkmen, Baluchis, Afghans, and Arabs living the county. The map of Iran, which shows the distribution of these ethnic groups, is available online [9]. These major ethnic groups are somehow different in their cultures, traditions, nutrition, and habits, which may predispose them to a diversity of CAD risk factors. In turn, they may experience different severity of cardiac diseases. There are a few studies having indicated differences in the risk factors among diverse ethnic groups [10, 11]. However, to the best of our knowledge, data on the distribution of the risk factors and the severity of CAD among various ethnic groups in Iran are scarce. Accordingly, we sought to assess the distribution of the well-known CAD traditional risk factors and the severity of CAD among major Iranian ethnic groups.

## Methods

### Design and Subjects

This was a retrospective study on 20,165 documented coronary artery disease patients who underwent coronary angiography at the Tehran Heart Center (THC) between 2011 and 2015. The THC is a tertiary heart hospital that serves patients with CAD referred from all around Iran. It is one of the largest cardiac hospitals in the Middle East. The angiography databank of the THC was utilized for the current study. The demographic, laboratory, clinical, and risk factor data of all patients who undergo coronary angiography at the THC are routinely collected by its trained physicians.

### Measures

Coronary artery disease and its severity were the main outcomes of the study. In the current study, a ≥50% luminal stenosis in individual epicardial vessels was considered coronary artery disease. In addition, the severity of the disease was assessed using the Gensini score. In 1983, Goffredo Gensini introduced this score to show the severity of coronary involvement [12]. This score assigns a severity score to each coronary stenosis according to the degree of the luminal obstruction and the location of the narrowing. According to this scoring system, reductions in the luminal diameter of 25, 50, 75, 90, and 99% as well as complete occlusion are given Gensini scores of 1, 2, 4, 8, 16, and 32, respectively. Every principal vascular segment is assigned a multiplier based on the functional importance of the myocardial area that this segment supplies. The left main coronary artery is assigned the significant multiplier × 5; the proximal segment of the left anterior descending coronary artery (LAD) × 2.5; the proximal segment of the circumflex artery × 2.5; the mid segment of the LAD × 1.5; the right coronary artery, the distal segment of the LAD, the posterolateral artery, and the obtuse marginal artery × 1; and all the other areas a factor of × 0.5. The more the Gensini score is, the higher is the severity of the disease. To interpret the data more precisely, based on the 33 and 67 percentiles of the patients’ Gensini scores, we categorized the scores into low (≤42), mid (>42 and ≤83), and high (> 83) Gensini score groups. Then, we assessed the distribution of the Gensini score categories in each ethnic group, too.

The main independent variable was ethnicity. In the present study, we considered self-reported Fars, Tork, Gilak, Mazani, Kord, and Lor groups as the major ethnic groups. Other minor ethnic groups (e.g., Turkmen, Baluchis, Afghans, Arabs, and Sistanis) were grouped as others.

The other independent variables (entered in the analysis as correlates) comprised the major modifiable risk factors (i.e., hypertension, diabetes mellitus, hyperlipidemia, smoking, male gender, positive family history of CAD, and obesity) as well as clinical and laboratory data.

### Statistical Analysis

The continuous variables are described with means and standard deviations (SDs), and the categorical variables are expressed as frequencies with percentages among the major ethnic groups. Adjusted standardized residuals were calculated for each ethnic group in a contingency table with the Gensini score groups. This measure is normally distributed under the assumption of independence between ethnic and Gensini score groups (the *χ* ^2^ test) and shows how far the observed frequencies in each cell are from their expected values. Also, the Gensini scores were compared between the ethnic groups using the Kruskal–Wallis test. Multiple comparisons were conducted based on ranks. To evaluate the association between the Gensini score and the ethnic groups, we applied a generalized linear model adjusting for potential confounders. Since the distribution of the Gensini score was skewed to the right, we employed the logarithm of the Gensini score instead, which showed a normal distribution. The statistical analyses were conducted using IBM SPSS for Windows, version 23.0 (Armonk, NY: IBM Corp), and a *P* value <0.05 was considered significant.

## Results

**Fig. 1:**
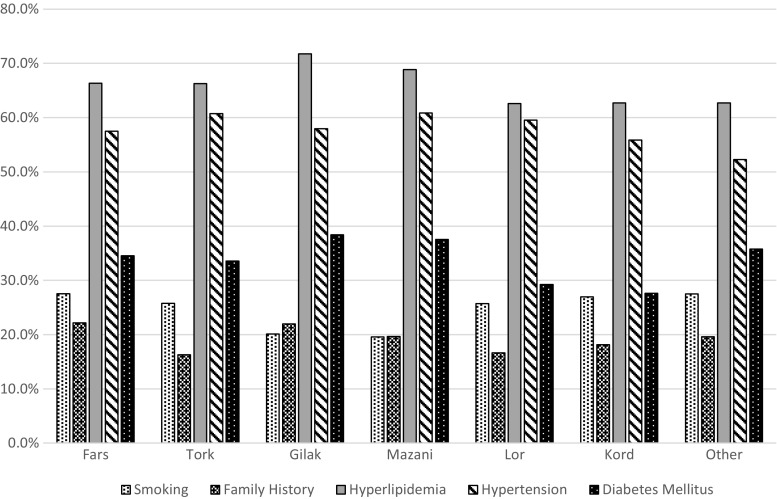
Distribution of different risk factors among ethnicities

In this study, 20,165 patients with documented coronary artery disease (14,131 [70.1%] men and 6034 [29.9%] women) at a mean age of 60.67 ± 10.79 years were recruited. Among the recruited patients, 59.8% were Fars, and 22.7, 5.9, 4.3, 3.4, and 2.6% were Torks, Gilaks, Mazanis, Lors, and Kords, respectively. The patients of the other minor ethnic groups accounted for 1.3% (262) of the study population. The distributions of age and sex among the participants were not considerably different. Table 1 illustrates the characteristics of the patient sample included in the study. In all the participants, hyperlipidemia was the most frequent risk factor (66.5%), followed by hypertension (58.4%), diabetes mellitus (34.3%), cigarette smoking (26.3%), and positive family history of CAD (20.4%). Figure 1 demonstrates the distribution of the risk factors among different Iranian ethnic groups.

**Table 1:** Characteristics of the recruited participants with documented coronary artery disease (*n* = 20,165)

	Ethnicity Groups							Total Patients		
	Fars (*n* = 12,062)	Tork (*n* = 4577)	Gilak (*n* = 1182)	Mazani (*n* = 876)	Lor (*n* = 687)	Kord (*n* = 519)	Others (*n* = 262)	Male	Female	Over All
Age (year)	60.21 ± 10.88	62.07 ± 10.59	60.66 ± 10.21	60.73 ± 10.57	60.67 ± 10.99	60.18 ± 10.55	58.61 ± 11.41	59.73 ± 10.99	62.88 ± 9.98	60.67 ± 10.79
Gender										
Male	8560 (71.0)	3203 (70.0)	767 (64.9)	556 (63.5)	490 (71.3)	375 (72.3)	180 (68.7)			14,131 (70.1)
Female	3502 (29.0)	1374 (30.0)	415 (35.1)	320 (36.5)	197 (28.7)	144 (27.7)	82 (31.3)			6034 (29.9)
Smoking	3315 (27.5)	1176 (25.7)	238 (20.1)	171 (19.6)	176 (25.7)	140 (27.0)	72 (27.5)	4809 (34.1)	479 (8.0)	5288 (26.3)
Family history	2648 (22.2)	736 (16.3)	257 (22.0)	170 (19.7)	114 (16.6)	93 (18.2)	51 (19.6)	2689 (19.2)	1380 (23.2)	4069 (20.4)
Hyperlipidemia	7929 (66.4)	2999 (66.3)	838 (71.7)	597 (68.9)	425 (62.6)	323 (62.7)	163 (62.7)	8602 (61.6)	4672 (77.9)	13,274 (66.5)
Hypertension	6937 (57.5)	2780 (60.7)	685 (58.0)	533 (60.8)	409 (59.5)	290 (55.9)	137 (52.3)	7187 (50.9)	4584 (76)	11,771 (58.4)
Diabetes mellitus	4162 (34.5)	1535 (33.6)	453 (38.4)	329 (37.6)	201 (29.3)	143 (27.6)	93 (35.8)	4016 (28.4)	2900 (48.1)	6916 (34.3)
Number of involved vessels										
1	3607 (29.9)	1225 (26.8)	258 (21.8)	233 (26.6)	227 (33.0)	149 (28.7)	78 (29.8)	3938 (27.9)	1839 (30.5)	5777 (28.6)
2	3706 (30.7)	1460 (31.9)	385 (32.6)	285 (32.5)	217 (31.6)	162 (31.2)	81 (30.9)	4490 (31.8)	1806 (29.9)	6296 (31.2)
3	4749 (39.4)	1892 (41.3)	539 (45.6)	358 (40.9)	243 (35.4)	208 (40.1)	103 (39.3)	5703 (40.4)	2389 (39.6)	8092 (40.1)
Recommendations										
Medical F/U	2670 (25.6)	962 (24.6)	247 (24.1)	160 (21.4)	129 (22.2)	115 (25.6)	57 (24.6)	2959 (24.3)	1381 (26.6)	4340 (25.0)
PCI	4572 (43.9)	1540 (39.4)	380 (37.0)	319 (42.6)	287 (49.4)	189 (42.0)	84 (36.2)	5280 (43.4)	2091 (40.3)	7371 (42.5)
CABG	3168 (30.4)	1406 (36.0)	400 (38.9)	269 (36.0)	165 (28.4)	146 (32.4)	91 (39.2)	3934 (32.3)	1711 (33)	5645 (32.5)
BMI (kg/m^2^)	27.55 ± 4.45	27.62 ± 4.44	27.39 ± 4.41	27.57 ± 4.64	27.11 ± 4.32	27.01 ± 4.17	26.92 ± 4.28	26.87 ± 4.02	29.07 ± 4.98	27.52 ± 4.44
EF (%)	47.32 ± 12.09	46.75 ± 23.62	47.68 ± 11.99	47.29 ± 12.21	46.97 ± 12.42	46.54 ± 12.91	46.16 ± 13.42	46.27 ± 16.76	49.23 ± 11.95	47.16 ± 15.53
Gensini score	72.59 ± 54.91	76.01 ± 55.77	77.09 ± 55.88	72.19 ± 50.42	67.47 ± 52.76	71.75 ± 59.04	70.98 ± 46.58	74.52 ± 55.17	70.77 ± 54.34	73.40 ± 54.95

Data are presented as mean ± SD or *n* (%)

*P* < 0.001

*F/U* follow-up, *PCI* percutaneous coronary intervention, *CABG* coronary artery bypass grafting, *BMI* body mass index, *EF* ejection fraction

As depicted in Table 1, the Fars ethnic group had the highest frequency of a positive family history of CAD. The Kords had the lowest rate of diabetes mellitus, and the Lors had the lowest prevalence of a positive CAD family history. Meanwhile, based on the Gensini score, the lowest severity of the disease was detected in the Lor ethnic group. The Mazani patients showed the lowest rate of cigarette smoking of all the ethnic groups, while the rate of diabetes was very high among them. The Torks constituted the only ethnic group with one of the highest Gensini scores, despite having a low frequency of CAD risk factors. The lowest prevalence of a positive family history of CAD was also found among the Tork patients. Among all the ethnic groups, the Gilaks had the worst risk factor profile insofar as they had the highest prevalence of hyperlipidemia, diabetes mellitus, and a positive family history of CAD. Still, the frequency of cigarette smoking was the lowest among the Gilaks. In addition, 3-vessel coronary artery involvement was also more frequently detected in the Gilaks than in the other ethnic groups. Accordingly, coronary artery bypass surgery was most frequently recommended for the Gilak patients. Apropos the severity of the disease, the Gilak ethnic group had the worst Gensini score.

The mean number of the risk factors was not clinically significant across the various ethnic groups: Fars 2.1, Torks 2.2, Gilaks 2.2, Mazanis 2.1, Lors 2.0, Kords 2.1, and others 2.1. Table 2 depicts the distribution of the number of risk factors among each ethnic group. As the table indicates, having two risk factors concurrently was the most frequent number of risk factors among all the ethnic groups. Also, this table shows that the Fars and Gilak ethnic groups had the highest prevalence of ≥4 simultaneous risk factors (8.7 and 8.6%, respectively). Meanwhile, the Lors and Kords showed the lowest rates (5.8 and 5.7%, correspondingly). Furthermore, having no conventional risk factor at all was found most frequently among the Lors (9.2%) and the Kords (8.9%).

**Table 2:** Distribution of the number of risk factors among different ethnic groups

Ethnic groups	Number of risk factors				
	0	1	2	3	≥4
Fars (*n* = 11,816)	820 (6.9)	2681 (2.7)	4125 (34.9)	3162 (26.8)	1028 (8.7)
Tork (*n* = 4459)	319 (7.2)	1028 (23.1)	1626 (36.5)	1193 (26.8)	293 (6.6)
Gilak (*n* = 1155)	80 (6.9)	252 (21.8)	401 (34.7)	323 (28.0)	99 (8.6)
Mazani (*n* = 855)	58 (6.8)	185 (21.6)	315 (36.8)	245 (28.7)	52 (6.1)
Lor (*n* = 676)	62 (9.2)	165 (24.4)	244 (36.1)	166 (24.6)	39 (5.8)
Kord (*n* = 507)	45 (8.9)	136 (26.8)	176 (34.7)	121 (23.9)	29 (5.7)
Other (*n* = 256)	21 (8.2)	71 (27.7)	76 (29.7)	68 (26.6)	20 (7.8)
Total (*n* = 19,724)	1405 (7.1)	4518 (22.9)	6963 (35.3)	5278 (26.8)	1560 (7.9)

Data are presented as *n* (%)

*P* < 0.001

The comparison of the coronary artery disease severity among all the ethnic groups is shown in Table 3. As the table illustrates, the distribution of the disease severity across the ethnic groups was significantly heterogeneous. According to Table 3, the Lors had the lowest severity of the disease as 41.3% of them had a Gensini score ≤ 42 (adjusted *R* 3.9). The lowest disease severity was found in the Tork ethnic group as 32.3% of them had a Gensini score ≤42 (adjusted *R* −3.3). Concerning the high severity of the disease, 35.2% of the Torks showed a Gensini score >83, which was the highest prevalence of all the ethnic groups (adjusted *R* 3.9). After the Torks, the Gilak ethnic group had the highest disease severity (36.0%, adjusted *R* 2.3). In contrast, the Far ethnic group showed the lowest severity of the disease (adjusted *R* −3.4) as 32.0% of them were in the high severity class (Gensini score >83), followed by the Lors (30.3%, adjusted *R* −1.5).

**Table 3:** Comparison of the coronary artery disease severity among all ethnic groups

Gensini score	Ethnic groups							
	Fars (*n* = 12,062)	Tork (*n* = 4577)	Gilak (*n* = 1182)	Mazani (*n* = 876)	Lor (*n* = 687)	Kord (*n* = 519)	Other (*n* = 262)	Total (*n* = 20,165)
≤42								
Count	4230	1478	371	299	284	182	83	6927
Percentage within each ethnic group	35.1	32.3	31.4	34.1	41.3	35.1	31.7	34.4
Adjusted residual	2.6	−3.3	−2.2	−0.1	3.9	0.3	−0.9	
>42 and ≤83								
Count	3978	1486	386	301	195	166	97	6609
Percentage within each ethnic group	33.0	32.5	32.7	34.4	28.4	32.0	37.0	32.8
Adjusted residual	0.8	−0.5	−0.1	1.0	−2.5	−0.4	1.5	
>83								
Count	3854	1613	425	276	208	171	82	6629
Percentage within each ethnic group	32.0	35.2	36.0	31.5	30.3	32.9	31.3	32.9
Adjusted residual	−3.4	3.9	2.3	−0.9	−1.5	0	−0.5	

*P* < 0.001

Based on the Kruskal–Wallis test, pairwise comparison between the different ethnic groups indicated that among all the ethnic groups, the severity of CAD was significantly different between the Torks and the Lors (*P* = 0.001), the Torks and the Fars (*P* < 0.001), the Gilaks and the Lors (*P* = 0.001), and the Gilaks and the Fars (*P* = 0.044).

The initial univariable analysis showed a significant (*P* < 0.001) association between the Gensini score and ethnicity (Table 1). To adjust the effect of possible confounders in this regard, we conducted a regression analysis (Table 4). As Table 4 shows, the results of the generalized linear model confirmed the univariable analysis inasmuch as, compared to the Fars ethnic group, the Gilaks had the worst severity (*β* 0.056, 95% CI 0.009 to 0.102; *P* = 0.018), followed by the Torks (*β* 0.032, 95% CI 0.005 to 0.059; *P* = 0.020). Meanwhile, the Lors showed the lowest severity (*β* −0.087, 95% CI −0.146 to −0.027; *P* = 0.004). For the other ethnic groups, the differences were not significant.

**Table 4:** Adjusted effect of ethnicity on cardiovascular disease severity based on the generalized linear model

Parameter	Coefficient	95% Wald confidence interval		*P*
		Lower	Upper	
Ethnicity groups				0.001
Fars	Baseline			
Tork	0.032	0.005	0.059	0.020
Gilak	0.056	0.009	0.102	0.018
Mazani	0.002	−0.051	0.055	0.933
Lor	−0.087	−0.146	−0.027	0.004
Kord	−0.038	−0.107	0.030	0.272
Others	0.001	−0.094	0.097	0.977
Male	0.128	0.103	0.154	0.000
Smoking	−0.0039	−0.065	−0.012	0.005
Family history	0.086	0.0059	0.113	<0.001
Hyperlipidemia	0.086	0.062	0.110	<0.001
Hypertension	0.004	−0.019	0.028	0.721
Diabetes mellitus	0.161	0.138	0.185	<0.001
Age	0.010	0.009	0.011	<0.001
BMI	−0.004	−0.007	−0.002	<0.001
EF	−0.011	−0.012	−0.010	<0.001
Intercept	3.818	3.703	3.934	<0.001

*BMI* body mass index, *EF* ejection fraction

## Discussion

The current study found heterogeneity in the severity of CAD and a diverse distribution in its well-known traditional risk factors among major Iranian ethnic groups. The severity of CAD among the Gilaks and Torks was significantly higher than that among the other ethnic groups and was notably lower in the Lor and Fars ethnic groups. Our results also revealed that the prevalence of having more than four risk factors simultaneously was significantly higher in the Fars and Gilak ethnic groups, while having no risk factor at all was more frequently seen among the Lors and the Kords. The Gilaks were the only group to exhibit the highest prevalence for three different risk factors (i.e., hyperlipidemia, diabetes mellitus, and a positive family history of CAD).

Previous studies, either in Western or in developing countries, have also demonstrated a disparity in CAD severity among diverse ethnical groups. Amin et al. [13] showed that whites and Asian Indians had a higher atherosclerotic burden than blacks and Hispanics, independent of risk factor diversity. Also, it has been found that Asian Indians have shorter telomeres, which may predispose this ethnic group to a higher prevalence of CAD (especially premature CAD) [14]. Moreover, it has been demonstrated that South Asians have greater risks for heart disease than Europeans and African-Caribbeans [15]. However, there are only a few studies that maintain an association between ethnicity and CAD. For instance, a study by Karlamangla et al. [16] on the quantification of socioeconomic status and ethnic differences in CAD risk in the USA indicated that this risk disparity was primarily related to socioeconomic status rather than ethnicity.

Iran is a multi-ethnic country that has always been the host of various ethnic groups in the past 4 millennia. The interpretation of Iranian social data is unsatisfactory, unless these ethnic groups are taken into consideration. Diverse cultures, traditions, habits, and nutrition among different Iranian ethnic groups may significantly influence the distribution and the severity of disease across them. Nevertheless, no precise information or formal national consensus report is available indicating the exact prevalence of diverse Iranian ethnic groups. Different reports have given the prevalence of the Fars ethnic group from 51 to 61%, Torks from 15 to 24%, Kords from 7 to 10%, Gilaks from 3 to 6%, Mazanis from 2 to 4%, and Lors from 3 to 5%. Additionally, the prevalence of the other ethnic groups, including Turkmen, Baluchis, Arabs, Afghans, Sistanis, Kormanjis, and Laris, has been reported to range from 5 to 10%, totally [17–19]. In our study, the prevalence of the studied ethnic groups was not considerably far from the previous findings within the literature.

The current study showed that the Gilaks were among the most vulnerable ethnic groups with a high prevalence of CAD risk factors and the most severe form of the disease. In addition, the Gilak ethnic group had the highest distribution of having more than four risk factors simultaneously as well as the highest prevalence of three conventional risk factors, which justifies the greater CAD severity. Gilaks are inhabitants of the northern Iranian province of Gilan, in the southern and southwestern coastal regions of the Caspian Sea. There is evidence showing that the ancestors of Gilak people came from the Caucasus region [20]. This evidence is somehow supported by some typological features of their language, which is shared with Caucasian languages [20]. A recent study [21] demonstrated a high frequency of Y-DNA haplogroups R1a (seen in vast areas of Eurasia, extending from Scandinavia, Central Europe, and southern Siberia to South Asia [22]), J2a (probably originated from the Caucasus Mountains region [23]), J1 (distributed in the Near East, Europe, the Caucasus, and Northeast Africa [24]), and G2a3b (mostly seen in the west of Russia, the Black Sea, the Middle East, and Iran [25]). The major occupations of Gilaks are fishing and agriculture. Gilan Province is one of the most attractive places in Iran and has a vibrant tourist industry during the summer. Inasmuch as fishing, agriculture, and tourism services are considered as seasonal jobs, many Gilak people are without work during particular periods of the year, which may predispose them to a less active and sedentary life. Furthermore, even though sea food is deemed the main source of food in Gilan, Gilaks are famous for their tasty foods, also rich in fat. This may result in hyperlipidemia, which was most frequently seen in our Gilak ethnic group (71.1%) as compared to the other ethnic groups. Furthermore, previous studies on the inhabitants of the northern parts of Iran have shown a low physical activity lifestyle and a considerably high rate of obesity and overweight in Gilaks, as one fifth to one fourth of them had a high body mass index [11, 26].

The Torks accounted for the second most vulnerable ethnic group in our study as they showed a high mean Gensini score. Moreover, the distribution of the Torks in the more severe CAD category (Gensini score >83) was significantly higher than that in the less severe CAD category (Gensini score ≤42) (adjusted *R* 3.9 vs. −3.3, respectively). Unlike our Gilak participants, there was no agreement between the level of the frequency of varied risk factors and the severity of CAD in the Tork ethnicity. Since none of the conventional risk factors had the highest frequency in the Torks and even a positive family history of CAD showed the lowest rate among this group, it seems that something other than the conventional risk factors is involved. This finding is supported by other studies having indicated an independent association between ethnicity and CAD and its severity, which cannot be explained by atherosclerotic risk factor profiles [5, 13, 27]. Torks are the second largest ethnic group in Iran. Even though they are widely distributed in the country, most Torks reside in the northwest of Iran, which is among the most densely populated regions. A recent study on the Y chromosome of Iranian Torks indicated a high level of gene diversity compatible with patterns registered in two neighboring countries, Turkey and Azerbaijan [28]. Based on data coming from Europe, the rate of CAD is 37.6% in Azerbaijan and 25.7% in Turkey [29] as compared to 19.4% [30] in the general population of Iran. Consequently, genetic factors may be behind CAD development and its severity among patients with Tork ethnicity.

In the present study, the lowest mean Gensini score was detected in the Lor ethnic group. Furthermore, by comparison with the other ethnic groups, the proportion of the Lors in the low Gensini score (≤42) category was high (adjusted *R* 3.9).

Having no CAD risk factor at all was seen in 9.2% of the patients in the Lor ethnic group, which was the highest rate of all the ethnic groups. Not only were the conventional risk factors not more frequent among the Lors, but also the Lors had the lowest rate of having a positive family history of CAD of all the ethnic groups. The majority of Lors live in the western and south-western parts of Iran. With respect to genetic background, the R1 group comprises the single most common haplogroup in the Lor ethnicity [21]. Lors show an elevated frequency of Y-DNA haplogroup R1b, which is the most frequently occurring paternal lineage in Western Europe, Russia, and Central Africa [30]. Several Lors live as tribes of herdsmen in the mountainous parts of Iran, migrating between summer and winter quarters. They are famous for their high daily physical activity and consumption of fresh natural foods. Their healthy lifestyle and nutritional habits are among the most important factors for CAD prevention.

The Fars ethnic group demonstrated the highest rate for a positive family history of CAD and the highest rate for having more than four simultaneous conventional risk factors. However, other than family history, the other conventional CAD risk factors were not prominently high among this ethnic group. Furthermore, the least proportion of this ethnic group was seen in the high Gensini score category (adjusted *R* −3.4). Therefore, besides the Lors, the Fars ethnic group showed the lowest vulnerability for the severity of CAD of all the ethnic groups. The Fars ethnic group is the most common ethnicity in Iran [17–19]. Although Fars are distributed all over the country, they commonly live in the central and northeast parts of Iran. The genetic background of the Fars ethnic group is characterized by an mtDNA (H and U haplogroups) pool composition, identical to people from Europe and Western Asia [31]. In Iran, even though the health system is distributed all over the country, most health facilities are located in the large cities, where the Fars ethnic group tends to live. This may result in a more convenient access to medical care for the Fars ethnic group than for the other ethnic groups. Psychosocial privileges of being in majority as well as better access to health services may explain the lower severity of CAD among this ethnic group.

### Strengths and Limitations

To the best of our knowledge, our study was the first of its kind to assess the association between the risk and severity of CAD among Iranian ethnic groups. The THC is a very large tertiary hospital rendering services to patients from all over Iran and has well-established quality-controlled databases with precise information on its admitted patients. Another strong point of this study is the number of recruited patients, which was large enough to include a significant number of patients from all the major ethnic groups. However, the current study was not without limitations. First of all, ethnicity was defined based on self-reported data, which can present some complexities. For instance, there are cross-ethnicity marriages, which may result in a mixture of different ethnic groups. Another shortcoming is that our study, albeit large in scale, was based on a referral hospital’s data and its results cannot be generalized to the whole population of Iran. Accordingly, other population-based studies drawing upon national or regional data are warranted. Moreover, due to cultural or political sensitivity to some ethnic groups, some individuals may have preferred to hide their own ethnicity and use other ethnicities instead. Fortunately, such sensitivity in Iran is negligible and this scenario is ordinarily unlikely, unless in very special circumstances. Another drawback of this study is that we did not consider the socioeconomic status of the patients simply because it was not available. Estimating the size of coronary artery stenosis was visually made by our cardiologists, and there was no instrument for measuring the size. This could be another limitation of the current study. Finally, we did not follow up our patients to evaluate their outcomes and to assess the prognosis of CAD in the different ethnic groups.

## Conclusions

This study found differences in the modifiable risk factors and severity of CAD among Iranian patients attending the THC between years 2011 and 2015. The highest prevalence rates of the risk factors and severity of CAD were found among the Gilak and Tork ethnic groups. Our results provide new insights into the role of ethnicity and its association with the major risk factors and severity of CAD in Iran and may, as such, help decision-makers and clinicians develop culturally sensitive interventions, prevention programs, and medical services particularly aimed at lessening CAD risk burden in vulnerable ethnic groups. Future longitudinal national and regional population-based studies are, however, warranted.
